# Financial challenges of providing obstetric services at rural US hospitals

**DOI:** 10.1111/jrh.70082

**Published:** 2025-10-21

**Authors:** Busse CE, O'Hanlon K, Kozhimannil KB, Interrante JD

**Affiliations:** ^1^ Division of Health Policy and Management University of Minnesota Minneapolis Minnesota USA; ^2^ Rural Health Research Center, Division of Health Policy and Management University of Minnesota Minneapolis Minnesota USA

**Keywords:** closure, finance, maternal health, obstetric, rural

## Abstract

**Purpose:**

This study describes perspectives of rural hospital administrators regarding the financial context for operating obstetric units, including the unique challenges they face and the strategies they have implemented to maintain obstetric services.

**Methods:**

In this mixed‐methods study, we used data from a survey we conducted from March to August 2021 of administrators of rural hospitals that had maintained or closed their obstetric units. Key financial outcomes included general finances, size and equipment, payor mix, workforce, and other fixed costs, examined descriptively. We also conducted thematic content analysis of open‐ended responses to financial questions.

**Findings:**

Respondents from hospitals that closed obstetric services (*n* = 40) reported that physician shortages (67%), financial losses (62%), clinical safety (56%), liability insurance costs (51%), and nurse shortages (39%) influenced the decision to close obstetric units. Among hospitals with obstetrics (*n* = 88), more than half (55%) reported that their hospital was operating with a profit margin, but only 41% said their obstetric unit had more revenue than costs. Of the hospitals with obstetrics who responded about the future of their obstetric units, 77% (61/79) were confident that they would continue providing obstetric care in 10 years; their open‐ended responses highlighted the importance of hospital leadership's commitment to maintaining obstetric services in their communities.

**Conclusions:**

Rural hospitals cite clinical workforce challenges, high fixed costs, and declining birth volumes as financial challenges to providing obstetrics. Strategies for maintaining obstetric care in rural communities should account for lower birth volumes in rural facilities and these interrelated challenges.

## INTRODUCTION

From 2010 to 2022, 238 rural hospitals closed their obstetric units,^1^ and financial concerns are a frequently cited reason for obstetric unit closures in rural communities.^2^ By 2022, nearly 60% of rural counties did not have hospital‐based obstetric services.^3^ In rural counties that are not adjacent to urban counties, loss of hospital‐based obstetric care is associated with increased risk of preterm birth and out‐of‐hospital births, and rural obstetric unit closures are associated with an increased risk of births occurring in a hospital without an obstetric unit—frequently, in an emergency department.^4^

Beyond service line closures, full hospital closures also affect rural communities. Since 2005, 196 rural hospitals have completely closed or stopped offering inpatient services.^5^ Hospitals that closed were more likely to have negative operating margins,^6^ lower levels of profitability, and lower patient volume in the years preceding closure compared with rural hospitals that remained open.^7^ Many remaining rural hospitals face financial stressors and market challenges. Compared with urban hospitals, rural hospitals have smaller operating margins^8^ and provide a higher percentage of uncompensated care.^9^ On average, inpatient volumes in rural hospitals are declining^10^ as rural populations are decreasing in size.^11^, ^12^ Further, some rural patients bypass their local hospital to seek care at larger hospitals,^13^, ^14^ particularly for elective surgical procedures, which are an important revenue source for many rural hospitals.^15^

Maintaining an obstetric unit can be an additional financial stressor for rural hospitals because of the high fixed costs of this service line. These costs include 24/7 staffing of a primary maternity care clinician (midwife, family physician, obstetrician‐gynecologist) and appropriately trained nursing staff, as well as specialized equipment for maternal and neonatal care (including equipment and protocols in place for emergency responses), operating room access, liability insurance, and ongoing investment in education, training, and quality improvement.^16^ In addition, the obstetric unit must have all personnel required to perform an emergency cesarean delivery readily available, including a physician with privileges and anesthesia providers.^16^

Hospitals are typically reimbursed for obstetric services through volume‐based payments, but hospitals incur fixed costs even when no births occur at their facility for days or weeks. As a result, hospitals that have low birth volumes struggle to maintain financially solvent obstetric units^2^ and are at an increased risk of closing their obstetric unit.^17^ Additionally, patients giving birth at rural hospitals are more likely to be covered by Medicaid insurance compared with those giving birth at urban hospitals: Medicaid covers 47% of births in rural areas compared with 40% in urban areas.^18^ Medicaid reimbursement rates for obstetric care are generally lower than rates of commercial insurers,^19^ which may make the mix of revenue from Medicaid and commercial insurers at rural hospitals financially consequential. Rural hospitals also face challenges recruiting and retaining obstetric staff, and in low birth volume settings, additional investment in training may be needed for providers to maintain clinical competence and confidence during rare, high‐risk, or emergency situations.^20^, ^21^ Although challenges around staffing and clinical safety are not exclusively caused by financial stress, the relationships between financial factors, staffing, and clinical safety at rural hospitals remain understudied.

Although prior research, described above, has revealed a range of issues that contribute to rural maternity care access challenges, there is limited evidence regarding the financial context rural hospitals navigate when they make decisions about obstetric service lines. To address this, we describe the perspectives of rural hospital administrators across the United States (US) about the financial considerations of operating obstetric units at rural hospitals, including the unique challenges they face and the strategies they have implemented to mitigate them.

## METHODS

This study used data from a 2021 survey of US rural hospitals that included questions about finances related to providing obstetric services. Obstetric service availability among all short‐term acute care hospitals in the US was identified using a previously published, validated method with data from the American Hospital Association Annual Survey, the Centers for Medicaid & Medicare Services Provider of Services file, and review of hospital websites and news stories.^22^ The survey frame included (1) a random 20% sample of hospitals with obstetric services in 2018 located in rural counties (all nonmetropolitan statistical areas^23^) with a majority non‐Hispanic White population; (2) all hospitals with obstetric services in 2018 in rural counties without a majority non‐Hispanic White population (“majority Black, Indigenous, People of Color”); and (3) all hospitals in rural counties that closed their obstetric units between 2010 and 2018. Administrators at 429 hospitals were surveyed between March and August 2021, and responses were received from 133 hospitals (31% response rate). We excluded five respondents because they did not answer financial questions critical to this analysis, resulting in a final sample size of 128 hospitals. The characteristics of survey respondents are reported in Table A1. This research was reviewed by the University of Minnesota institutional review board and designated as exempt.

Hospitals that had closed their obstetric units were asked a separate set of questions in the survey than those with obstetric services. Among those that had closed their obstetric units, the survey asked about factors that influenced closure decisions, including financial losses, workforce shortages and challenges, clinical safety concerns, liability insurance costs or concerns, hospital mergers or acquisitions, and other financial and nonfinancial concerns.

Among hospitals with obstetric services, we examined a variety of hospital characteristics and factors related to the financial operations of rural obstetric units. We used data from the 2018 American Hospital Association Annual Survey to examine critical access hospital status (which allows hospitals to receive cost‐based reimbursement for Medicare services^24^), census region, average daily census, and the percent of inpatient days that were Medicaid funded. Rural county type (micropolitan, noncore) and urban adjacency was measured using 2013 Urban Influence Codes^25^ and county racial and ethnic proportions were calculated using data from the American Community Survey.^26^ All other variables came from the survey (Table A2). These included questions around key financial outcomes that we examined within five broad categories: (1) general finances—whether the hospital and obstetric unit were operating with a profit margin; (2) hospital size, services, and equipment— number of inhospital births, service sharing to reduce costs, operating room dynamics, and items needed to provide safer obstetric care; (3) payor mix (the percentages of revenue from different types of health insurers used to finance patient care)— payors for hospital births and impacts on hospital financial viability; (4) clinical workforce— ability to offer competitive salaries, funding, and protected time available to train obstetric clinicians; (5) other fixed costs— liability insurance and challenges from other fixed costs. Questions about the number of inhospital births and whether the hospital and specific service lines were operating with a profit margin asked about 2019 (prior to the COVID‐19 pandemic). We also examined other obstetric service factors that may impact obstetric service line planning, including whether the respondent was confident their hospital would continue to provide obstetric services in 10 years, changes in annual births in the last 3 years, obstetric nonmedical bypass rate, pregnant patient referral rates, and distance to the next‐nearest hospital with obstetric services.

We conducted a descriptive analysis, with categorical variables reported as frequency and percent and continuous variables described using median and interquartile range (IQR). Among hospitals with obstetric services, we stratified survey responses by whether the respondent was confident that their hospital would continue to provide obstetric services in 10 years. We also examined whether responding hospitals differed from nonresponders (Table A3). Quantitative analyses were conducted using SAS 9.4.

The survey included several open‐ended questions, encouraging respondents to provide additional details about their hospital (Table A4). Respondents’ answers to the closed‐ended questions determined when they were prompted to provide an open‐ended explanation. CB and KO extracted all responses to open‐ended questions pertaining to finance and conducted a thematic content analysis.^27^ CB and KO independently reviewed these responses for familiarization and collaboratively developed a deductive coding strategy for each question. They independently coded the open‐ended responses, including interpreting latent content, and inductively added codes as needed. They met to discuss areas of disagreement and finalized coded responses.

## RESULTS

Of the 128 hospital respondents in 2021 in the analytic sample, 88 (69%) reported that their hospital provided obstetric services, and 40 (31%) reported that their hospital had stopped providing obstetric services. In this sample, 35% of hospitals that maintained obstetric services were critical access hospitals compared with 73% of hospitals that stopped providing obstetric services (Table A5). Responding and non‐responding hospitals did not differ significantly with respect to population characteristics and hospital finances, size, and payor mix, but responding hospitals were more likely than non‐responding hospitals to be from the Midwest and Western regions of the United States (Table A3).

### Hospitals that closed obstetric units

Respondents from hospitals that closed their obstetric units reported that physician shortages (67%), financial losses (62%), clinical safety (56%), and liability insurance costs (51%) influenced the hospital's decisions to close the obstetric unit (Table 1). Less frequently cited reasons for closure included nurse shortages (39%), scheduling challenges (26%), other financial concerns (15%), other nonfinancial concerns (8%), and hospital mergers (5%).

**TABLE 1:** Factors that influenced closure among hospitals that closed obstetric services (*n* = 40).

Factors that influenced closure	*N* (%)^a^
Physician shortage	26 (66.7)
Financial loss	24 (61.5)
Clinical safety concerns associated with too few births	22 (56.4)
Liability insurance costs or concerns	20 (51.3)
Nurse shortage	15 (38.5)
Scheduling challenges	10 (25.6)
Other financial concerns^b^	6 (15.4)
Other nonfinancial reasons^c^	3 (7.7)
Hospital merger or acquisition	2 (5.1)

^a^ Proportion among the 39 respondents who answered this closed‐ended survey question. The one respondent who did not answer this closed‐ended survey question answered the related open‐ended survey question, “To the best of your knowledge, why did the hospital stop offering inpatient labor and birth services?”, so they were not excluded from the analysis.

^b^ Respondents elaborated in the open‐ended answers that their other financial reasons were: cost of full‐time anesthesia and operating room staff (*n* = 1), high percentage of Medicaid births (*n* = 2), lack of surgical services (*n* = 1), cost of 24/7 availability of anesthesia service (*n* = 1), and maintenance requirements/costs (*n *= 1).

^c^ Respondents elaborated in open‐ended answers that their other nonfinancial reasons were: cesarean delivery backup (*n* = 1), provider call schedules (*n* = 1), and patient preferences for obstetricians (not family practice providers) (*n* = 1).

All respondents from hospitals that closed their obstetric units also described the reasons for closure in open‐ended answers. Half (*n* = 20) specifically stated that clinical workforce shortages were a key factor in their closure decision, six of whom elaborated that their hospital had just one clinician attending births prior to closure or that they made the decision to close after the departure of a single key staff member. One respondent explained, “We only had one provider delivering babies and it became difficult with 24/7 call and time away.” Other key factors influencing closure that respondents shared in their open‐ended answers were challenges of providing care in facilities with low birth volumes, specifically financial losses (*n* = 17); challenges meeting clinical requirements and certifications (*n* = 10), including performing a cesarean delivery within 30 min and maintaining full‐time anesthesia coverage; and difficulties maintaining clinical skills with low birth volumes (*n* = 9). Acknowledging the interrelated nature of these factors, one respondent wrote, “the low volume of births made the [obstetric] service financially and operationally non‐viable.”

### Hospitals that maintained obstetric services

Among the 88 hospitals with obstetric services in the analytic sample, 35% were designated as critical access hospitals (Table 2). More than half of respondents in hospitals with obstetric services (55%) reported that their hospital was, as a whole, operating with a positive profit margin, but just 41% of their obstetric units had more revenues than costs.

**TABLE 2:** Financial characteristics and related resources of hospitals with obstetric services (*n* = 88).

Financial characteristics	*N* (%)^a^
**General finances**	
Critical access hospital^b^	31 (35.2)
Hospital operating with a profit margin in 2019^c^	
Yes	45 (54.9)
No	19 (23.2)
Do not know	18 (22.0)
Obstetric unit operating with a profit margin in 2019^d^	
Yes	33 (40.7)
No	29 (35.8)
Do not know/other	19 (23.5)
**Hospital size, services, and equipment**	
Average daily census, median (IQR)^b^	22 (10, 53)
Number of inhospital births in 2019, median (IQR)	272 (119, 445)
Type of operating room	
Main hospital/general operating room	45 (51.1)
Dedicated operating room for obstetric services	41 (46.6)
Do not perform cesarean deliveries	2 (2.3)
Hospital shares services to reduce fixed costs of obstetrics^e^	48 (62.3)
Hospital lacks services, equipment, or facilities that would help to provide safer care during labor and birth^f^	33 (37.9)
**Payor mix**	
Percent of hospital inpatient days funded by Medicaid, median (IQR)^b^	18.1 (11.9, 22.8)
Percent of births at hospital paid by Medicaid, median (IQR)^g^	62.0 (40.0, 80.0)
Payor mix affects financial viability^d^	22 (27.2)
**Workforce**	
Able to offer competitive clinician salaries^d^	51 (63.0)
Hospital offers funding to train labor and birth clinicians (obstetricians, midwives, and family medicine physicians)^h^	32 (43.8)
Hospital offers protected time to train labor and birth clinicians (obstetricians, midwives, and family medicine physicians)^h^	26 (35.6)
Hospital offers funding to train labor and birth nurses^i^	55 (68.8)
Hospital offers protected time to train labor and birth nurses^i^	45 (56.3)
**Other fixed costs**	
Cost of liability insurance impacts obstetric services^j^	17 (21.8)
Other fixed costs make it difficult to financially maintain the obstetric service line^j^	23 (29.5)

*Note*: IQR = interquartile range (quartile 1, quartile 3).

^a^ Missing responses are not included in the column percentages.

^b^ Data from the 2018 American Hospital Association Annual Survey.

^c^ Proportions among the 82 respondents who answered this survey question.

^d^ Proportion among the 81 respondents who answered this survey question.

^e^ For example, obstetrics sharing anesthesia services with general surgery, obstetrics sharing ultrasound with radiology. Proportion of the 77 respondents who answered this survey question.

^f^ Proportion of the 87 respondents who answered this survey question.

^g^ Proportion of the 83 respondents who answered this survey question.

^h^ Proportion of the 73 respondents who answered this survey question.

^i^ Proportion of the 80 respondents who answered this survey question.

^j^ Proportion of the 78 respondents who answered this survey question.

Respondents’ hospitals had an average daily census of 22 [IQR 10, 53] and 272 births annually [IQR 119, 445]. Slightly less than half of hospitals (47%) had a dedicated operating room for obstetric services, and slightly more than half (51%) used the main hospital operating room for obstetric services. The majority of respondents (62%) reported that their hospital shared resources across service lines to reduce the fixed costs of obstetrics, like sharing anesthesia and ultrasound with other departments. Summarizing this cost‐conscious approach, one respondent wrote, “we share as many of our resources as possible within the hospital to reduce costs.”

In addition, 38% of respondents with obstetric services reported that their hospital lacked services, equipment, or facilities that would help them provide safer care during labor and birth, and each offered additional context in open‐ended responses. Some respondents focused on changes related to staffing (*n* = 13) or facilities (*n* = 8) that would allow the hospital to provide more timely care or to reduce the need to transfer patients needing a higher level of care. Many (*n* = 16) listed equipment needs, which ranged widely in scope and cost; examples included monitoring systems (*n* = 10), “one or two updated newborn warmers,” “birthing beds that work as they should,” and a “postpartum hemorrhage cart.” Several respondents (*n* = 7) described a desire to provide wraparound services for their patients, including “breastfeeding support groups [and] services for those struggling with substance abuse,” prenatal classes, telehealth options, increased postpartum support, and mental health resources.

Respondents’ hospitals varied in the prevalence of Medicaid‐paid births and the impact of payor mix on hospital finances. A median of 18.1% of hospitals’ inpatient days were funded by Medicaid (IQR 11.9%, 22.8%). In contrast, 62.0% of births were paid by Medicaid (IQR 40.0%, 80.0%). Just over one quarter of respondents (27%) said that payor mix affects the financial viability of their obstetric service line. Of the 13 respondents who provided additional detail in open‐ended answers, all described low Medicaid reimbursement rates compared to commercial insurance as a challenge, and several (*n* = 5) noted a high proportion of their obstetric patients were insured by Medicaid. Respondents explained that obstetric units must have either a certain number of patients with commercial insurance or a particular volume of births “to stay afloat financially.”

Financial challenges in maintaining hospitals’ obstetric care workforce were common. Although 63% of respondents representing hospitals with obstetric services reported that their hospital was able to offer competitive clinician salaries, in open‐ended answers, respondents explained that recruiting clinicians goes beyond simply offering an attractive salary: “often providers desire more of an urban environment.” Sometimes, hospitals used creative strategies to encourage retention and recruitment of obstetric clinicians. One respondent shared that their hospital had a “new physician recruitment endowment” and another explained, “our pay is not competitive with larger, urban centers, so we emphasize a lifestyle choice.” Another stated plainly, “Hospital wide, our salary scale is woefully below the area average.” Although more than half of respondents reported that their hospital offered funding (69%) or protected time (56%) to train nurses in obstetrics skills, these financial training components were less often reported for hospitals’ obstetricians, midwives, and family physicians (44% reported funding and 36% reported protected time for these clinicians).

Other fixed costs of operating obstetric units were also frequently noted financial challenges. One fifth (22%) of respondents said that the cost of liability insurance impacts their obstetric service line, whereas one third (30%) reported that other fixed costs also made it difficult for them to maintain their obstetric units. In their open‐ended answers, one respondent simply wrote, “all costs matter,” and three respondents cited the high cost of liability insurance as the reason that their hospital does not offer trial of labor after cesarean delivery or vaginal birth after cesarean. Another shared that some of their providers stopped providing obstetric care because “liability insurance was costing more than the pay they got for deliveries.”

### Predictions about providing obstetric care in 10 years

Survey respondents from hospitals providing obstetric care were asked to predict whether they were confident that their hospital would continue to provide obstetric care in the next 10 years. Of the 79 respondents who answered the question, 61 (77%) stated they were confident that their hospital would continue to provide obstetric services in the next 10 years, whereas 18 (23%) were unsure or said that their obstetric services were likely to close in the next 10 years (Table 3). Many respondents (*n* = 50) explained the factors informing their prediction in a subsequent open‐ended question. In their answers, respondents detailed concerns around financial viability (*n* = 7), declining birth volumes in their communities (*n* = 9), and clinician recruitment (*n* = 9). Two respondents described having just one clinician to attend deliveries at their hospital, and another wrote, “[providing obstetric services] is a discussion every month and if we can't attract more providers, then we will not be able to sustain.” Several respondents (*n* = 11) highlighted the important role hospital leaders play in the decision to maintain obstetric services, with one writing, “The senior team and board of directors do not want to take services away from our community if at all possible” and another explaining, “Our facility's board of directors is dedicated to providing obstetric services to members of the community where they live and work. Obstetric services [are] an important aspect of community health.” Relatedly, many (*n* = 23) also highlighted the importance of providing obstetric services in their communities, sharing that “more patients are staying in town to deliver instead of traveling to one of the ‘bigger’ hospitals” and “as long as this is a county‐funded hospital, we will continue delivering babies because, honestly, there is nowhere else for these families to go.”

**TABLE 3:** Characteristics of hospitals by whether they are confident they will be providing obstetric care in 10 years (*n* = 79).

	Will you be providing obstetric care in 10 years?	
Characteristics	Confident yes *N* (%)^a^ *N* = 61	Other (likely to close and unsure) *N* (%)^a^ *N* = 18
**County characteristics**		
County type^b^		
Micropolitan, adjacent	16 (26.2)	9 (50.0)
Micropolitan, nonadjacent	24 (39.3)	3 (16.7)
Noncore, adjacent	9 (14.8)	4 (22.2)
Noncore, nonadjacent	12 (19.7)	2 (11.1)
Majority Black, Indigenous, People of Color^c^	11 (18.0)	4 (22.2)
**General finances**		
Critical access hospital^d^	17 (27.9)	9 (50.0)
Hospital operating with a profit margin in 2019^e^		
Yes	34 (56.7)	9 (50.0)
No	14 (23.3)	4 (22.2)
Do not know	12 (20.0)	5 (27.8)
Obstetric unit operating with a profit margin in 2019^f^		
Yes	26 (44.1)	6 (33.3)
No	18 (30.5)	8 (44.4)
Do not know/other	15 (25.4)	4 (22.2)
**Hospital size, services, and equipment**		
Average daily census, median (IQR)^d^	22 (12, 60)	27 (8, 49)
Number of inhospital births in 2019, median (IQR)	334 (130, 470)	190 (90, 262)
Hospital shares services to reduce fixed costs of obstetrics	36 (64.3)^g^	8 (47.1)
Hospital lacks services, equipment, or facilities that would help to provide safer care during labor and birth	22 (36.1)	8 (44.4)
**Payor mix**		
Percent of hospital inpatient days funded by Medicaid, median (IQR)^d^	17.6 (12.1, 22.4)	20.3 (18.1, 25.3)
Percent of births at hospital paid by Medicaid, median (IQR)	55.0 (35.0, 80.0)	72.5 (60.0, 80.0)
Payor mix affects financial viability	16 (27.1)^h^	4 (22.2)
**Workforce**		
Able to offer competitive clinician salaries	41 (69.5)^h^	8 (44.4)
**Other fixed costs**		
Cost of liability insurance impacts obstetrics	11 (18.6)^h^	5 (27.8)
Other fixed costs make it difficult to financially maintain the obstetric service line	13 (22.0)^h^	9 (50.0)
**Other obstetric service factors**		
Change in annual births in last 3 years		
Increased	9 (14.8)	1 (5.6)
Decreased	30 (49.2)	13 (72.2)
Stayed the same	22 (36.1)	4 (22.2)
Obstetric nonmedical bypass rate		
<10%	22 (36.1)	2 (11.1)
10%–24%	18 (29.5)	4 (22.2)
25%–50%	10 (16.4)	6 (33.3)
>50%	3 (4.9)	3 (16.7)
Don't know	8 (13.1)	3 (16.7)
Pregnant patients referred out of hospital to higher level of care		
≥10%	20 (32.8)^i^	9 (50.0)^j^
Distance to next‐nearest hospital with labor and birth services		
>60 mi	15 (24.6)	8 (44.4)

*Note*: IQR = interquartile range (quartile 1, quartile 3).

^a^ Missing responses are not included in column percentages.

^b^ Categorized using 2013 Urban Influence Codes from the American Community Survey.

^c^ Categorized using county racial and ethnic proportions from the American Community Survey.

^d^ Data from the 2018 American Hospital Association Annual Survey.

^e^ Proportions for the “confident yes” column among the 60 respondents who answered this survey question.

^f^ Proportions for the “confident yes” column among the 59 respondents who answered this survey question.

^g^ Proportion of the 56 respondents who answered this survey question.

^h^ Proportion of the 59 respondents who answered this survey question.

^i^ *n* = 1 respondent (1.6%) answered “don't know.”

^j^ *n* = 2 respondents (11.1%) answered “don't know.”

Among the 61 respondents who were confident their hospital would provide obstetric care in 10 years, 57% said their hospital was operating with a profit margin overall, and 44% said their obstetric unit had more revenue than costs. Among the 18 who said their hospital was likely to close the obstetric unit or that they were unsure, 50% said their hospital was operating with a profit margin, and 33% of obstetric units had more revenues than costs.

Hospitals confident they would continue obstetric services had a similar average daily census as those likely to close or unsure if services would continue. However, compared to hospitals likely to close or unsure if services would continue, those confident they would continue obstetric services more often reported sharing services to reduce obstetric costs (64% vs. 47%), less often reported lacking services, equipment, and facilities to provide safer childbirth care (36% vs. 44%), had less births funded by Medicaid (55% vs. 73%), a higher proportion said payor mix affected their hospital's financial viability (27% vs. 22%), and a higher proportion reported being able to offer competitive clinician salaries (70% vs. 44%).

Among the other obstetric service factors that we examined by anticipated continuation of obstetric services, respondents at hospitals likely to maintain obstetric services less often reported that the annual number of births in their communities decreased in the last 3 years (49% vs. 72%), reported having 10% or more of obstetric patients bypass their hospital for nonmedical reasons (51% vs. 72%), referred 10% or more of their pregnant patients out of hospital to a higher level of care (33% vs. 50%), and reported that the next‐nearest hospital with labor and birth services was over 60 mi away (25% vs. 44%), compared with respondents who said their hospital was likely to close the obstetric unit in the next 10 years or that they were unsure.

## DISCUSSION

In this mixed‐methods study of the financial aspects of maintaining or closing obstetric units at rural hospitals, hospital administrators described interrelated challenges regarding clinical workforce shortages, high fixed costs of obstetric services, and declining birth volumes, and shared strategies they use to mitigate these challenges. The majority of respondents from hospitals that closed their obstetric units reported that physician shortages, financial losses, clinical safety, and liability insurance costs influenced their closure decision. Among respondents from hospitals with obstetric services, three quarters were confident that they would continue to provide obstetric services in 10 years; their open‐ended responses revealed the importance of hospital leadership's commitment to maintaining obstetric services in their communities.

The high fixed costs of providing obstetric services paired with volume‐based reimbursements are a major financial challenge for maintaining obstetric units at rural hospitals. This study indicated that financial shortfalls frequently preceded obstetric unit closures, with three in five respondents whose hospitals closed their obstetric units citing financial loss as a factor that influenced that decision. Regardless of birth volume, hospitals must pay for appropriate staff, equipment, and facilities to maintain obstetric services.^16^ Fewer than half of responding hospitals with obstetrics had a positive profit margin. To efficiently manage high fixed costs, two thirds of respondents at hospitals currently providing obstetric services reported sharing services, like anesthesia and ultrasound, with other units in their hospital. In addition to high fixed costs, almost one third of rural hospitals providing obstetric care also reported facing challenges related to payor mix. As Medicaid generally reimburses obstetric services at lower rates than commercial insurance^19^ and rural communities have a higher proportion of Medicaid‐paid births compared with urban areas,^18^, ^28^ this payor imbalance may further exacerbate challenges in covering fixed costs for rural hospitals. Changes to Medicaid policies or funding levels could influence whether rural hospitals decide to provide obstetric services, given the importance of Medicaid as a payor for childbirth‐related care. Payment strategies tailored to the needs of rural hospitals with low and variable obstetric volumes, like standby payments and low‐volume payment adjustments, may contribute to financial solvency for obstetric services by addressing fixed cost burdens and payor mix.^29^, ^30^

Clinical safety concerns stemming from low birth volumes and associated resource limitations were another major factor that led to rural obstetric unit closures, cited by more than half of respondents as a reason their hospital stopped providing obstetric care. Even among rural hospitals that maintained obstetric services, approximately one third reported that they lacked certain services, equipment, or facilities that would allow them to provide safer care during labor and birth, ranging from equipment needs like a postpartum hemorrhage cart, monitoring systems, an updated newborn warmer, and functioning birthing beds, to wraparound services like breastfeeding support groups and mental health resources. Among hospitals that did not report being confident their hospital would continue to provide obstetric services, this percentage was higher, with more than two fifths lacking these factors needed for safer care. At these same hospitals, three quarters of respondents reported a decline in births along with many local obstetric patients seeking care at nonlocal hospitals, further exacerbating the low birth volume financial and safety challenges. Funding and time for clinical staff to pursue continuing education and training are critical for maintaining skills, yet fewer than half of hospitals providing obstetric services in this study reported that they offered funding or protected time to train obstetric clinicians. Simulations, innovative staffing models, obstetric readiness training, and provider‐to‐provider telehealth services are some strategies that could facilitate continuing obstetric medical education and skill building for rural clinicians;^30^, ^31^ these, too, require time and financial support. Additionally, efforts to improve clinical safety should recognize the relationship between birth volume and outcomes as patients at lower birth volume rural hospitals have higher risks of severe maternal morbidity and mortality.^32^ Rural hospital administrators seem aware of these realities and would likely welcome policy efforts to help them overcome safety challenges related to low birth volume.

Lacking maternity care clinicians to provide obstetric services was another major reason listed by rural hospital administrators for the closure of their obstetric units; two thirds of them cited physician shortages, and more than one third separately cited nursing shortages. For many, loss of a single key provider forced them to close their obstetric unit. Less than half of rural hospitals in this study that were likely to close their obstetric unit in the future were able to offer competitive clinician salaries, but that proportion was higher at rural hospitals likely to maintain services. In this study, across rural hospitals with obstetric services, open‐ended responses highlighted that recruiting and retaining clinicians to live and practice in rural areas can be very challenging. Some rural hospitals, however, have met this challenge with success. For example, a critical access hospital in Kansas with approximately 300 births per year, built a thriving maternity care practice that attracts patients from a multi‐county area, recruiting high‐quality staff using a mission‐driven, service‐oriented mindset.^33^ Similarly, an analysis from Wisconsin found that meaningful work and integration in the local community were key factors influencing whether physicians in rural family medicine training programs were retained in rural practices.^34^ There is emerging interest in building academic‐rural partnerships to routinely incorporate rural rotations into training programs, which may help to expand the rural physician workforce.^35^

All of these factors—financing, safety, and workforce—are interrelated and inextricably linked to birth volume, which has long been substantially lower in rural and less densely populated US communities.^36^ Birth volume impacts the resources that rural obstetric units have, and consideration of birth volume is important in payment policies, quality improvement programs, and training, recruitment and retention of maternity care clinicians in rural communities.^29^, ^36^, ^37^ Previous research suggests strategies to address the financial challenges that rural hospital administrators face, including low‐volume payment adjustments, standby capacity payments, support for training and professional development of clinicians, and further development of regional and state networks such as perinatal quality collaboratives.^29^, ^31^, ^38^, ^39^

### Limitations

This study offers novel insight into rural hospitals’ financial challenges in providing obstetric services using data from a survey designed specifically for rural hospitals. However, the study findings should be interpreted in light of several limitations. The survey was administered during the COVID‐19 pandemic and had a response rate of 31%. Responding hospitals were generally similar to those that did not respond along most characteristics, except for US region, where responders were more often from the West and less often from the South (Table A3). Some of the financial factors that we examined had relatively high nonresponse from survey respondents (e.g., 17% for “hospital offers funding to train labor and birth providers” and “hospital offers protected time to train labor and birth providers”). Nine respondents did not answer the question asking how confident they were that their hospital would be providing obstetric services in 10 years, which was a main stratifying variable in this analysis. We attempted to reduce nonresponse by including the option “too difficult to predict at this time”; however, it is likely that respondents’ knowledge of hospital challenges, finances, and the ability to predict future decisions about service lines varied considerably by the respondent's role at the hospital (e.g., nurse manager, chief nursing officer, clinical leadership, chief executive officer, and administrator) and by the length of time they had worked at the hospital (ranging from 30% for 0–5 years to 32% for over 20 years; Table A1). In addition, this study focused on financial considerations for providing obstetric services at rural hospitals and did not include in‐depth survey questions about related challenges, like workforce recruitment and retention. Finally, rural obstetric unit and rural hospital closures have continued since 2021, when these survey data were collected, and it is important to continually collect and report information on rural obstetric financing as policy changes occur.

## CONCLUSION

Maintaining hospital‐based obstetric services in rural communities requires understanding the specific financial challenges and the broad financial context that rural hospitals face. In this mixed‐methods study, we analyzed closed‐ and open‐ended responses from a survey of rural US hospitals to understand the perspectives of rural hospital administrators regarding the financial aspects of operating obstetric units in their communities. We found that high fixed costs, safety concerns, and clinical workforce challenges are key financial obstacles for successfully operating obstetric units at rural hospitals. Strategies for maintaining obstetric care in rural places require attention to the ways in which lower birth volumes in rural hospitals shape their overall finances.

## CONFLICT OF INTEREST STATEMENT

The authors declare no conflicts of interest.

## Appendix

**TABLE A1:** TABLE Characteristics of survey respondents (*n* = 128).

Respondent characteristics	*N* (%)
Position/Title	
Nurse manager, L&B	67 (52.3)
Chief nursing officer	26 (20.3)
Clinical leadership	18 (14.1)
Chief executive officer	9 (7.0)
Administrator	4 (3.1)
Chief medical officer	1 (0.8)
Medical director	1 (0.8)
Head/Chief of obstetrics	1 (0.8)
Staff nurse	1 (0.8)
Highest level of education	
Some college	3 (2.4)
2‐year degree	19 (15.0)
4‐year degree	63 (49.6)
Masters	35 (27.6)
Doctorate	5 (3.9)
Doctor of Medicine (MD)	2 (1.6)
Missing	1
Years worked at current institution	
0–5	37 (28.9)
6–10	24 (18.8)
11–20	26 (20.3)
>20	41 (32.0)

*Note*: Percents do not all sum to 100% due to rounding.

**TABLE A2:** Finance‐related closed‐ended survey questions.

Survey questions for hospitals that closed their obstetric units
Are you aware of any of the following being a factor in the hospital's decision to stop offering inpatient labor and birth services? (please select all that apply): Financial losses associated with too few births Workforce shortages: physicians Workforce shortages: nurses Scheduling challenges Clinical safety concerns associated with too few births Liability insurance costs or concerns Hospital merger or acquisition Other financial concerns, please explain Other nonfinancial reasons, please explain
**Survey questions for hospitals with obstetric services**
*General finances*
Was your hospital operating in the Black (revenues exceeding costs) in 2019? (Yes; No; Other, please explain)
Was your obstetric service line/labor and birth unit operating in the Black (revenues exceeding costs) in 2019? (Yes; No; Other, please explain)
*Hospital size, services, and equipment*
Approximately how many births occurred in your hospital in **2019**?
Does your hospital offer services that reduce the fixed costs of providing your obstetric line? For example, general surgery services, which share anesthesia services, or ultrasound services in radiology? (Yes, please explain; No)
For cesarean deliveries, does your hospital use an operating room that is dedicated to obstetric cases or a general operating room that is also used for non‐obstetric cases? (Dedicated operating room for obstetric cases; Main hospital/general operating room; We do not perform cesareans (please explain why); Other, please explain)
Are there any services, equipment, or facilities your hospital lacks that would help to provide safer care during labor and birth? (No; Yes, please explain)
*Payor mix*
Approximately what percentage of the births at your hospital is paid for by… (Medicaid; Private or commercial insurance; Self‐pay or uninsured; Indian Health Service; Tricare; other)
Does your payor mix affect the minimum number of births necessary for financial viability? (Yes, please explain; No)
*Clinical workforce*
Are you able to offer competitive clinician salaries that encourage retention and recruitment of obstetric personnel? (Yes; No; Other, please explain)
Does your hospital offer funding and/or protected time to train labor and birth staff each year? (Checkbox matrix: Funds for training; Protected time for training; No training offered vs. providers (obstetricians, midwives, family physicians); RNs)
*Other fixed costs*
Is the cost of medical liability insurance making it difficult to financially maintain the obstetric service line? (Yes, please explain; No)
Are there other fixed costs making it difficult to financially maintain the obstetric service line? (Yes, please explain; No)
*Other obstetric service factors*
Assuming no changes in your community needs, workforce, or reimbursement, what is your prediction that you'll be providing inpatient labor and birth services at your hospital in 10 years? (Confident we will be offering inpatient labor and birth services; It is likely that our inpatient labor and birth services will close; Too difficult to predict at this time)
Has the annual number of births changed in the last 3 years? (Increased; Decreased; Stayed the same)
What percentage of pregnant patients do you refer out of your hospital to a higher level of care for delivery? (<10%; 10%–24%; 25%–50%; >50%; I don't know)
What is your bypass rate? i.e., What percentage of local pregnant patients chooses to give birth at nonlocal hospitals (more than 30 min away) **for nonmedical reasons**? (<10%; 10%–24%; 25%–50%; >50%; I don't know)
How far from your hospital is the next‐nearest hospital that provides inpatient labor and birth services? (<10; 10–29; 30–60; >60 mi)

**TABLE A3:** Comparison of responding and non‐responding hospitals (*n* = 429).

Characteristic	Responded (*n* = 128)	No response (*n* = 301)^a^	*p*‐value^b^
**Location**			
Region, *n* (%)^c^			0.02
Northeast	6 (4.7)	26 (8.6)	
Midwest	48 (37.5)	102 (33.9)	
South	37 (28.9)	118 (39.2)	
West	37 (28.9)	55 (18.3)	
County type, *n* (%)^d^			0.38
Micropolitan, adjacent	33 (25.8)	101 (33.6)	
Micropolitan, nonadjacent	36 (28.1)	69 (22.9)	
Noncore, adjacent	30 (23.4)	62 (20.6)	
Noncore, nonadjacent	29 (22.7)	69 (22.9)	
County racial/ethnic majority Black, Indigenous, People of Color, *n* (%)^e^	20 (15.6)	55 (18.3)	0.51
**Hospital finances, size, payor mix**			
Critical access hospitals, *n* (%)^c^	60 (46.9)	113 (37.5)	0.07
Average daily census, median (IQR)^c^	18 (7, 46)	17 (8, 47)	0.92
Hospital control, *n* (%)^c^			0.56
Government, nonfederal	35 (27.3)	76 (25.3)	
Government, federal	3 (2.3)	16 (5.3)	
Nongovernment, not‐for‐profit	75 (58.6)	171 (56.8)	
For‐profit	15 (11.7)	38 (12.6)	
Percent of hospital inpatient days funded by Medicaid, median (IQR)^c^	18.1 (9.7, 25.0)	17.2 (9.1, 23.3)	0.54

^a^ *n* = 5 respondents who were excluded from the analytic sample because they did not answer key financial questions central to this analysis.

^b^ *p*‐values are Chi‐square tests for categorical variables and Wilcoxon rank‐sum tests for continuous variables.

^c^ From the 2018 American Hospital Association Survey.

^d^ From the 2018 Area Health Resource File.

^e^ From the 2018 American Community Survey.

**TABLE A4:** Finance‐related open‐ended survey questions.

Survey questions for hospitals that closed their obstetric units	Responses
To the best of your knowledge, why did the hospital stop offering inpatient labor and birth services?	40 people asked, 40 responses
**Survey questions for hospitals with obstetric services**	**Responses**
Assuming no changes in your community needs, workforce, or reimbursement, what is your prediction that you'll be providing inpatient labor and birth services at your hospital in 10 years? Please explain your answer above	93 people asked, 50 responses
Does your hospital offer services that reduce the fixed costs of providing your obstetric line? For example, general surgery services, which share anesthesia services, or ultrasound services in radiology?—Yes, please explain	48 people asked, 35 responses
Are you able to offer competitive clinician salaries that encourage retention and recruitment of obstetric personnel?—Yes—Text	51 people asked, 8 responses
Are you able to offer competitive clinician salaries that encourage retention and recruitment of obstetric personnel?—No—Text	14 people asked, 3 responses
Are you able to offer competitive clinician salaries that encourage retention and recruitment of obstetric personnel?—Other, please explain	16 people asked, 16 responses (many noninformative)
Is the cost of medical liability insurance making it difficult to financially maintain the obstetric service line?—Yes, please explain	16 people asked, 15 responses (many noninformative)
Are there other fixed costs making it difficult to financially maintain the obstetric service line?—Yes, please explain	22 people asked, 21 responses (many noninformative)
Does your payor mix affect the minimum number of births necessary for financial viability?—Yes, please explain	22 people asked, 15 responses
Are there any services, equipment, or facilities your hospital lacks that would help to provide safer care during labor and birth?—Yes, please explain—Text	33 people asked, 33 responses

**TABLE A5:** Descriptive characteristics of hospitals who responded to the survey by whether the hospital maintained or closed the obstetric unit (*n* = 128).

Hospital characteristics	Maintained obstetric unit *N* (%) (*N* = 88)	Closed obstetric unit *N* (%) (*N* = 40)
Critical access hospital^a^	31 (35.2)	29 (72.5)
County type^b^		
Micropolitan, adjacent	27 (30.7)	6 (15.0)
Micropolitan, nonadjacent	29 (33.0)	7 (17.5)
Noncore, adjacent	17 (19.3)	13 (32.5)
Noncore, nonadjacent	15 (17.1)	14 (35.0)
Region^a^		
Northeast	5 (5.7)	1 (2.5)
Midwest	26 (29.6)	22 (55.0)
South	26 (29.6)	11 (27.5)
West	31 (35.2)	6 (15.0)
Majority Black, Indigenous, People of Color^c^	15 (17.1)	5 (12.5)
Average daily census in 2018, median (IQR)^a^	22 (10, 53)	9 (4, 37)

*Note*: IQR = interquartile range (quartile 1, quartile 3).

^a^Data from the 2018 American Hospital Association Annual Survey.

^b^Categorized using 2013 Urban Influence Codes from the American Community Survey.

^c^Categorized using county racial and ethnic proportions from the American Community Survey.
